# Examination of self patterns: framing an alternative phenomenological interview for use in mental health research and clinical practice

**DOI:** 10.3389/fpsyg.2024.1390885

**Published:** 2024-07-09

**Authors:** Anya Daly, Rosa Ritunnano, Shaun Gallagher, Laurence J. Kirmayer, Nicholas Van Dam, Joshua Kleinman

**Affiliations:** ^1^Department of Philosophy, School of Humanities, University of Tasmania, Tasmania, TAS, Australia; ^2^Centre for Youth Mental Health, Faculty of Medicine, Dentistry, and Health Sciences, University of Melbourne, Parkville, VIC, Australia; ^3^Institute for Mental Health, School of Psychology, University of Birmingham, Birmingham, United Kingdom; ^4^University of Memphis, Memphis, TN, United States; ^5^University of Wollongong, Wollongong, NSW, Australia; ^6^Division of Social and Transcultural Psychiatry, Department of Psychiatry, McGill University, Montreal, QC, Canada; ^7^Culture & Mental Health Research Unit, Lady Davis Institute, Jewish General Hospital, Montreal, QC, Canada; ^8^Contemplative Studies Centre, School of Psychological Sciences, University of Melbourne, Melbourne, VIC, Australia; ^9^College of Medicine, The Ohio State University, Columbus, OH, United States

**Keywords:** mental health, lived experience, pattern theory of self, phenomenological psychopathology, examination of self-patterns, mental disorders, phenomenological interview, nosology and classification of mental disorders

## Abstract

Mental disorders are increasingly understood as involving complex alterations of self that emerge from dynamical interactions of constituent elements, including cognitive, bodily, affective, social, narrative, cultural and normative aspects and processes. An account of self that supports this view is the pattern theory of self (*PTS*). The *PTS* is a non-reductive account of the self, consistent with both embodied-enactive cognition and phenomenological psychopathology; it foregrounds the multi-dimensionality of subjects, stressing situated embodiment and intersubjective processes in the formation of the self-pattern. Indications in the literature already demonstrate the viability of the *PTS* for formulating an alternative methodology to better understand the lived experience of those suffering mental disorders and to guide mental health research more generally. This article develops a flexible methodological framework that front-loads the self-pattern into a minimally structured phenomenological interview. We call this framework ‘Examination of Self Patterns’ (*ESP*). The *ESP* is unconstrained by internalist or externalist assumptions about mind and is flexibly guided by person-specific interpretations rather than pre-determined diagnostic categories. We suggest this approach is advantageous for tackling the inherent complexity of mental health, the clinical protocols and the requirements of research.

## 1 Background

Early in the 20th century, Jaspers (1913, 1963) cautioned about the inadequacies of the fixed categorical approaches to psychopathology along with the related ambitions to establish third person, objective measures in psychiatry comparable to the hard sciences. Categories and classifications, Jaspers suggested, are useful fictions in the context of psychiatric assessment, and they offer only a provisional guide to the clinician. While most clinicians and researchers would disagree with the notion that such classifications are ‘fictions’ in the strong sense, many would agree that current psychiatric categories and classifications are pragmatic heuristics at best (Hyman, 2010; Heckers, 2015; Kendler, 2016). Despite acknowledgement of the limitations of diagnostic categories, the enterprise of consistent classification has tended toward the reification of diagnoses even with the lack of association with objective markers (Hyman, 2010). In response, some major funding bodies have shifted their approach to dimensional frameworks (Cuthbert, 2022). Arguably, however, these approaches have themselves become reified alongside the diagnoses; further emphasising the reliance on popular or preferred biological units of explanation and largely ignoring much of what goes on at the level of the clinician and the individual experiencing distress (Weinberger et al., 2015; Kendler, 2024).^1^ The reification of categories and biophysiological units of analysis is in large part responsible for the well-documented problems associated with the *DSM* and to a lesser degree the *ICD* which fail to adequately take account of the intrinsically shifting and interconnected nature of psychopathology, thereby eluding “diagnostic order” (Jaspers, 1913, 1963; Maj, 2005; Nordgaard et al., 2013; Stanghellini and Broome, 2014; Daly and Gallagher, 2019).

Despite the various evolutions of diagnostic assessment aimed at addressing the evaluative deficiencies of the structured checklist approach (Pilgrim and Bentall, 1999; Fulford et al., 2005; Andreasen, 2007; Decker, 2013; Paris, 2013; Parnas and Gallagher, 2015), and the purported aim of inclusivity with the biopsychosocial model, many deficiencies in representing the experiential reality of mental disorders persist. Moreover, the emphasis within the research funding structure and popular clinical training models largely remains on objective means and measures (Scharfstein, 2005; Read et al., 2009, 2011). As Parnas and Gallagher note: “The psychiatric object is typically portrayed as an objective, thing-like entity, unproblematically graspable as it exists ‘in itself’ through a behaviorist third-person perspective and as being indicative of a specific and modular physiological dysfunction” (Parnas and Gallagher, 2015, p. 65). Importantly, however, although few approaches fully embrace the ‘psychiatric subject’, often constraining discussion of subjective experience to pre-existing domains and categories, these long-standing debates have recently spurred incorporation of broader conceptualisations of mental disorders; for example, in culture-related considerations and in the adoption of a lifespan approach (Reed et al., 2019) and in the reconceptualization of mental disorder as a complex, dynamic system with symptoms that will change over a lifetime (Scheffer et al., 2024).

With the aim of addressing some of these shortcomings, the contemporary phenomenological project has been advocating, for at least the past 20 years, a return to subjectivity and the experiential realm as the fundamental pre-requisite for psychopathological assessment and treatment (Broome et al., 2013; Parnas et al., 2013; Stanghellini et al., 2019; Broome, 2020). During this period, several phenomenological semi-structured interviews have been developed for use by researchers and clinicians. These interviews enable the exploration of alterations of various aspects of experience in mental disorders^2^—with a more or less explicit diagnostic purpose depending on the specific interview. The targeted aspects of experience include, among others: disorders of basic or “minimal” self-awareness (*EASE*, Parnas et al., 2005); disorders of the lived world (*EAWE*, Sass et al., 2017); the psychopathology of imagination (*EAFI*, Rasmussen et al., 2018); abnormal features of lived temporality (*TATE*, Stanghellini et al., 2022a).

More generally, the phenomenological interview (Høffding and Martiny, 2016) has been used to investigate various dimensions of psychopathology. With respect to depression, for example, although some of these dimensions are already documented in the *DSM*-5 and *ICD*-11, phenomenological interviews have highlighted significant aspects of symptomatology in depression that have been overlooked, revealing the complex network of meanings in narratives along with the dynamical relations between self-related processes (Kirmayer et al., 2017). Concurrently, new approaches have been developed to integrate philosophical phenomenology with qualitative research to ground the focus of a qualitative study. One such approach is *Phenomenologically Grounded Qualitative Research* (*PGQR*, Køster and Fernandez, 2021), which draws on phenomenological “existentials” such as selfhood, temporality, spatiality, and affectivity to inform qualitative data analysis and study design.

Despite such developments, the field of phenomenological psychopathology, with a few exceptions [e.g., Pienkos (2020)], has tended to focus on isolated and decontextualized anomalous experiences. This focus comes at the expense of more situated investigations that consider factors such as world events, personal meanings, painful affect, interpersonal, cultural, and intersectional aspects of self-experience, normative factors, autobiographical narratives, social and historical conditions affecting the emergence of psychopathology. In response to this, there have been calls to develop new, ethically responsive and engaged phenomenological approaches to explore the embedded and situated quality of psychopathology (Kirmayer, 2015; Ritunnano, 2022; Stanier, 2022; Pienkos et al., 2023; Spencer and Broome, 2023). These approaches give attention to the personal, agentive, interpersonal, and socio-cultural factors that shape the experience of mental illness or disability. They also take account of epistemic and hermeneutical asymmetries within the research encounter, addressing the ways in which the experience of illness may be de-contextualized or stripped of its meaning within unilateral research agendas. In clinical settings, however, practitioners using phenomenological interviews are more disposed to recognize that experience is always lived through constellations of relations within rich social and cultural contexts, rather than as a depoliticized and ahistorical abstraction. With this in mind, phenomenologically informed clinicians can play a transformative role in addressing hermeneutic forms of injustice, even within the constraints of the clinical encounter. With the right tools, clinicians attuned to recognizing subjective experience as inherently valuable can go a long way toward better capturing the reality of mental disorders (potentially yielding new information immediately relevant to clinical assessment and treatment) and can also help shift the mental health research agenda away from the dominant focus on reductionist approaches that, despite considerable investment, have failed to result in clinically actionable outcomes (Insel, 2022).

## 2 A novel framework: examination of self patterns

The current organization of mental health care in terms of discrete diagnostic categories has attracted much criticism from both outside and within the profession of psychiatry. Despite the widely recognized problems with current nosologies, psychiatrists must still ‘work around’ the deficiencies of the well-entrenched system to protect the interests of their clients and those under their care to ensure access to medications, welfare, insurance, and basic services. Nevertheless, there have been recurrent calls for a paradigm shift that would privilege patients experience, focus on dynamic processes, and engage with the context of the patient’s lifeworld (Bracken et al., 2012; Rose and Rose, 2023; Scheffer et al., 2024). Our current proposal responds to such calls by providing important theoretical and methodological foundations for a novel approach to phenomenological interviewing. We draw on a comprehensive theoretical framework that supports this view: the pattern theory of self (*PTS*) (Gallagher, 2013, 2024; Gallagher and Daly, 2018; Daly and Gallagher, 2019). The *PTS* is a non-reductive account of the self, consistent with embodied-enactive approaches to cognition and consonant with views in phenomenological psychopathology dating back to the work of Karl Jaspers. The *PTS* takes a pluralist, holistic approach, defining the self as a pattern of dynamically related processes that include cognitive, bodily, affective, social, narrative and normative factors or processes intertwined in dynamical relations. The *PTS* foregrounds the multi-dimensionality of the subject, stressing both situated embodiment and the significance of intersubjective processes in the formation of the self-pattern. Importantly, it emphasizes the connectivity and dynamic relations among the diverse processes of the self-pattern and offers a way to track not only the origins, complexities and progression of mental disorders but also therapeutic transformations of the individual toward a meaningful life with robust self-esteem and positive connections to community.^3^ We propose that many psychiatric disorders can be understood as disruptions in the self-pattern.^4^ These disruptions arise in particular contexts and in response to specific challenges that can be revealed through a *minimally-structured phenomenological interview* – the *ESP.*

In the following sections, we provide both the theoretical foundation and some practical guidelines for implementing the *ESP* for research purposes, as well as for gaining deeper insights into the lived experience of individuals experiencing mental distress. In this paper, we do not provide a standardized measure of the experience of the self-pattern. However, we do discuss interview strategies and some illustrative examples of how such a measure could be developed (see section 6 below). These suggestions may prove useful for researchers developing an interview schedule tailored for their particular study aims. An interview protocol for exploring the ESP framework is currently being developed further for use in clinical and non-clinical populations.

The *ESP* shares a key theoretical commitment of phenomenological interview methodologies widely discussed in the literature (Parnas et al., 2005; Petitmengin, 2006; Høffding and Martiny, 2016; Sass et al., 2017; Rasmussen et al., 2018; Køster and Fernandez, 2021; Stanghellini et al., 2022a; Frohn and Martiny, 2023) in eschewing physicalist reductionism, exemplified by both the medicalized disease model and the more recent neurocentric view. The *ESP* distinctively provides a rethinking of the “psychiatric object,” in favor of an understanding of the multi-dimensionality of the individual dynamically immersed in a meaningful shared world that can be captured in the 10 elements/ processes of the self-pattern. Rather than providing a symptom checklist or a scale-based diagnostic tool, the *ESP* builds on some of the strategies and nuances found in the various semi-structured interviews from phenomenological psychiatry and qualitative approaches grounded in phenomenological philosophy.^5^ It also coheres well with more recent approaches such as those detailed in Reed et al. (2019) and Scheffer et al. (2024).

This article first presents the evolution of a theory that has contributed to this novel approach. This is followed by an explanation of the concept of self-pattern, examining 10 contributing factors and showing how these factors are interrelated and display implicative interdependencies. We also suggest how one of these factors, narrative capacity, reflects and to varying extents discloses all the others. Finally, we detail how this framework can be “frontloaded” into phenomenological interview techniques to support “a rich diagnosis” in the clinical setting (Parnas and Gallagher, 2015), and to generate research data suitable for both qualitative analysis and quantitative analysis.^6^

As commonly understood, in the process of obtaining a psychopathological description, researchers and clinicians face the challenge of translating the person’s experience (as lived in the first-person perspective) into specific signs and symptoms that are defined in the third-person. This translation aims to produce an “objective” account that can reliably contribute to classification for research, diagnosis, and treatment purposes (Parnas et al., 2013). While there has been a return to the experiential realm through the use of semi-structured and semi-qualitative phenomenological interview designs, and the intersubjective endeavor of psychopathology research has been emphasized (Galbusera and Fellin, 2014), nonetheless, such approaches still retain the end goal of assigning a diagnostic category that can inform clinical treatment, identify targets for social interventions, and facilitate access to appropriate resources. As Nordgaard et al. (2013, p. 354) describe it: “The goal of a psychiatric assessment is to describe the patient’s complaints, appearance, and existence in an actionable psychopathological format, namely, one that results in diagnostic classification and other clinical decisions.” It is important to note that diagnostic classification is only one aspect of an assessment which includes identifying the patient’s problems, predicaments, and concerns (Mezzich et al., 2010). These all contribute to a clinical formulation that can guide a treatment plan with potentially multiple types or levels of intervention. Much like in case formulation [see, e.g., Bieling and Kuyken (2003)], diagnostic classification, is *not* the primary aim of the *ESP* approach. Nonetheless, the *ESP* can potentially contribute to both the development of better diagnostic nosology by revealing salient dimensions of experience that may have been neglected as well as better clinical assessment by foregrounding the salient experiences and concerns of the patient. The *ESP* invites researchers to follow a ‘minimally structured’ approach to phenomenological interviewing to gain a more in-depth and nuanced understanding of the self and its alterations. This can lead to a clinically useful formulation of the patient’s problems. In some instances, this approach could be sufficient to identify the issues relevant to specific research questions or for clinical intervention. It might also indicate the need to follow-up with a more directed interview as detailed in the various semi-structured interviews of phenomenological psychiatry. Notably, this approach shifts the emphasis from concerns with operationalisation of constructs, measurability of experience and external reliability/validity, to focus on lived experience with significant epistemic responsibility accorded to the patient in co-directing the interview.

To summarize, the ESP can lead to a clinically useful formulation of the patient’s problems by: (1) employing a more comprehensive conception of the self that is based on the PTS which is ‘front-loaded’ into the interview; (2) revealing salient dimensions of lived experience that may be neglected by other interview schedules or aproaches that are organized in terms of a checklist of symptom criteria; (3) according more epistemic responsibility to the patient in the interview process through self-selecting salience; (4) recognising and supporting a co-constructed narrative approach in the interview. These strategies help to ensure that the interview remains person-centred and that it can map more precisely the aspects of experience that are causing suffering and difficulties in the person’s engagement with others and in everyday functioning.

## 3 The pattern theory of self: the theoretical basis for the *ESP*

The *PTS* rejects the accounts in the history of philosophy and psychology that depend on the “assumption that ‘self’ is a persisting, unified and transparent locus of experience and that factors external to the embodied self are contingent and adventitious, whereas factors internal to the embodied self [or even brain] are more essential and definitive” (Daly and Gallagher, 2019, p. 6). That is, the *PTS* challenges the assumptions of a pure interiority of consciousness/self and pure exteriorities of others and world, and further embraces a plurality of aspects that contribute to a sense of self that is dynamically unfolding (Gallagher and Daly, 2018). According to *PTS*, the concept of “self” comprises a pattern of elements or processes, none of which on their own is sufficient to identify any particular self. Instead, the self-pattern operates as a complex system that emerges from dynamical interactions of constituent elements that include aspects of the individual and the relationships in which they are embedded. While we will not delve into all the philosophical intricacies that this proposal entails, for example, questions about necessary and sufficient conditions, continuity over time, personal identity, and the nature of the minimal self (some of these issues are addressed in Gallagher, 2024 and Gallagher and Daly, 2018). For the purposes of this discussion, Table 1 provides a list of elements that constitute the self-pattern, along with some notes about the dynamical relations that integrate these elements into a gestalt. This information should be sufficient background for understanding the *ESP*.

**Table 1:** Elements of the self-pattern [reproduced with the permission of Gallagher (2024)].

Elements of the pattern	Brief description
Bodily processes	Includes core bio-systemic and autopoietic processes related to motoric, autonomic, endocrine, enteric, immune, interoceptive functions, allowing the overall system to maintain homeostasis necessary for survival, and to distinguish between itself and what is not itself.
Prereflective experiential processes	Includes prereflective self-awareness, a structural feature of first-person consciousness constrained by bodily factors; the sense of ownership (mineness) and the sense of agency, which can involve various sensory-motor modalities, such as proprioception, kinaesthesia, touch and vision. These aspects form the experiential core of what is sometimes called the minimal self.
Affective processes	The fact that someone manifests a certain temperament or emotional disposition reflects a particular mix of affective factors that range from very basic and mostly covert or tacit bodily affects (e.g., hunger, fatigue, libido) to what may be a typical emotion pattern, a set of existential feelings, a background mood.
Behavioral/action processes	Behaviors and actions make us who we are – behavioral habits and skills reflect, and perhaps actually constitute, our character. This is a classic view that goes back at least to Aristotle.
Social/intersubjective processes	Humans (possibly some non-human animals) are born with a capacity for attuning to intersubjective existence; at a certain point in social relations a more developed self-conscious recognition of oneself as being distinct from others, a sense of self-for-others, and a sense of being part of a group or community.
Cognitive/psychological processes	These are aspects emphasized in traditional theories of personal identity highlighting psychological continuity and memory, including one’s conceptual understanding of oneself, beliefs, cognitive dispositions, as well as personality traits.
Reflective processes	The ability to reflect on one’s experiences and actions – closely related to notions of autonomy and moral personhood, including the capacity to reflectively evaluate and form second-order volitions about one’s desires.
Narrative processes	Self-interpretation has a narrative structure and recursively reflects (and often reinforces) the self-pattern. On some theories, selves are inherently or constitutively narrative entities.
Ecological processes	We tend to identify ourselves with our stuff -- physical pieces of property, clothes, homes, and various things that we own, the technologies we use, the institutions we work in, etc. Our embodied-situated actions engage with (and sometimes incorporate) artifacts, instruments, bits and structures of the environment in ways that define us and scaffold our identities. Situations shape who we are, and affordances define our possibilities.
Normative processes	Our extensive engagement with the environment includes social and cultural practices. These are not just what we do, but involve what we ought to do, and obligations that we keep or not. Constraints (and sometimes well-defined roles) imposed by social, cultural, institutional factors shape our habitual behaviors, and our self-conceptions of who we are, and who we think we should be.

Importantly, we emphasize that the dynamical nature of the self-pattern is such that any particular process is to be considered dynamically interwoven with the other processes. Failure to emphasize this has led to a misunderstanding of *PTS*, as if it provides a mere list of aspects without accounting for how they interrelate (de Haan et al., 2017) or fails to explain how they integrate into an explanatory whole (Kyselo, 2014). Nonetheless, de Haan et al. (2017) rightly suggest that “in the case of psychiatric disorders and their treatment the relevant questions precisely pertain to this structure, to the relation between aspects of the self. On the one hand, it’s not enough to simply say that the self-pattern is a dynamical gestalt,” and that the different elements of the self-pattern are dynamically related de Haan et al. (2017, pp. 5–6). On the other hand, this is not something that one can determine *a priori*, or simply by adopting a particular theory. Rather, we need empirical and clinical studies to help specify how something like the dynamical integration of a self-pattern can occur, how it is ordered and how it can become disordered.

Dynamic relations characterize all of the processes involved in the self-pattern, including embodied, enactive and intersubjective processes. This also applies to neural processes. We acknowledge that the brain may produce multiple mappings of changes in the self-pattern and brain research may reveal specifically how first-person experience is reflected in a material system that can be observed and analyzed from a third-person point of view. Fingelkurts and Fingelkurts (2017), for example, suggest the value of the *PTS* is precisely in its capacity to flexibly correlate with and map changes in neurophysiology. More relevant to the current project, narrative functions in a similar way in that once experience is articulated, it becomes available to third parties for understanding (or misunderstanding), analysis and evaluation.^7^

## 4 The disclosure of self-patterns through narratives

The *PTS* includes narrative as one of the key elements of the self-pattern, but it also suggests that self-narrative can reveal and track changes in the self-pattern more generally. The individual self-narrative is not only one element in the self-pattern but may reflect aspects of the other processes and elements in the self-pattern, including intersubjective and normative factors.^8^ In this regard it is able to play a hermeneutical role in so far as it facilitates an integration of the other elements of the self-pattern to aid sense-making and self-understanding [see Gallagher (2024)]. In the context of the *ESP*, the patient’s self-narrative, as told to the therapist, thus plays a dual role in *giving access* to subjective experience and *revealing the changing self-pattern* (and the dynamic relations amongst the factors of the self-pattern) of the patient/interviewee. Self-narrative both encapsulates what has happened – events, experiences and histories – and also reveals the narrator in the telling. Narratives reflect the changing perspective and self-experience of the patient/interviewee, and this is likely to vary from day to day, according to both the experiences that impact the patient/interviewee and the relational context within which the interview is conducted. Predictably, this contingency and dynamism are especially evident with people grappling with anomalous experiences or mental health challenges.^9^

As previously emphasized, the self-pattern is dynamic, evolving or changing in response to interventions, ongoing experience and understanding. These changes can be tracked in the narratives of the patient but are also reflected in the narratives of the clinician, the family and significant others in interaction with the patient. The *ESP* aims to facilitate the co-construction of a narrative (specifically, a self-narrative for the patient) as well as the discovery of previously occluded narratives which may serve to further illuminate the challenges being faced by the patient. When applied in clinical settings, the *ESP* approach may support the creation of aspirational narratives pointing in the direction of possible treatment pathways. In research settings, as proposed above, these narratives offer a dynamical mapping of the self-pattern which can be used, for instance, to understand the effects of treatment (such as in the case of *DBS*; Dings and de Bruin, 2016; Gilbert and Viaña, 2018).

In earlier works, Gallagher and Daly (2018) and Daly and Gallagher (2019) provided a comparative list and table to illustrate the advantages of using the *PTS* to identify elements of symptomatology not captured by the *DSM* in cases of Major Depressive Disorder. These were extracted from narratives of patients with lived experience of depression through a variety of interviews, biographies, vignettes, and also from narratives embedded in websites of institutions and associations dedicated to supporting those with depression. It is important to note that the *DSM* consulted at that time has changed; it is now more inclusive; for example, giving more recognition to social, political and cultural factors in mental health, and emphasizing dimensional features that allow that symptoms are not “on–off” phenomena; they manifest in degrees of intensity and severity, and change over time. Nonetheless, despite all the evolutions of the *DSM*, it continues to attract criticism not only from outside the psychiatric profession, but also from leading figures within the profession.^10^ What remains relevant to our current project is the claim that the adoption of the *ESP* framework for the assessment of psychopathology in clinical or research contexts has distinctive advantages.^11^

This claim is supported by the recent review article, “The lived experience of depression: a bottom-up review co-written by experts by experience and academics” (Fusar-Poli et al., 2023). This broad survey of first-person accounts (across several cultures and languages) documenting the lived experience of depression focused on “experiences consistent with the *DSM/ICD* diagnostic criteria/requirements for unipolar depression,” derived phenomenologically informed themes from an extensive review of qualitative studies that included first-person reports of experience. The themes correlate well with the self-pattern factors and were confirmed by “experts by experience,” i.e., by those who had experienced depression, including some of the 28 co-authors drawn on by Fusar-Poli et al. (2023).

The subjective world of depression was characterized by an altered experience of emotions and body (feeling overwhelmed by negative emotions, unable to experience positive emotions, stuck in a heavy aching body drained of energy, detached from the mind, the body and the world); an altered experience of the self (losing sense of purpose and existential hope, mismatch between the past and the depressed self, feeling painfully incarcerated, losing control over one’s thoughts, losing the capacity to act on the world; feeling numb, empty, non-existent, dead, and dreaming of death as a possible escape route); and an altered experience of time (experiencing an alteration of vital biorhythms, an overwhelming past, a stagnation of the present, and the impossibility of the future)…. [As well as] by altered interpersonal experiences (struggling with communication, feeling loneliness and estrangement, perceiving stigma and stereotypes) [that] varied across different cultures, ethnic or racial minorities, and genders (Fusar-Poli et al., 2023, p. 352).

One can discern elements of the self-pattern in these first-person narratives. The following table (Table 2) offers a few brief examples to show the correlations between themes and self-pattern processes. As Fusar-Poli et al., note, these narratives “are not assumed to represent entirely distinct categories”; they interconnect and often overlap, which, again, may indicate how these processes are dynamically intertwined. “For example, while we sought to distinguish between mental and physical experiences of depression, first-person narratives do not clearly differentiate between the bodily and the mental domains” (Fusar-Poli et al., 2023, p. 353).

**Table 2:** Correlation of examples of first-person narrative statements of major depression disorder and the self-pattern.

**Self-pattern processes**	**First-person narratives** – the excerpts below are from Fusar-Poli et al. (2023); they cohere well with the earlier table (Daly and Gallagher, 2019) drawing on the PTS.
Bodily Processes	“I am tired in the morning and tired at night and tired all day and never, never feel fresh.” “I had sleep problems … I had poor appetite. I was constipated …. I also had back pain and sexual problems.” “I do everything automatically, the signals from my body are shut down, I do not listen, I become like a machine, just doing what needs to be done.”
Pre-reflective experiential	“There was no real connection. You feel like you are talking and doing everything you should, but you are not really there. It’s like you are removed from yourself ….” “All I seemed to be able to do was exist in the moment with no drive or purpose, no reason for being.”
Affective	“I had a fear of change, fear of dying, fear of failure, fear of success, fear of being alone, which paralyzed me for years and years.” “I get angry. I just hate noise. It disturbs and destroys me, and I find myself arguing with others.” “A loss of feeling, a numbness, had infected all my human relations. I did not care about love, my work, family, friends …. or physical/ emotional intimacy …. I was losing myself, and that scared me.”
Behavioral/ action	“At first you can still kind of function in the world – but then …. you start living in your own mind.” “I never know whether I’m gonna be able to do what I planned that day until I get up that morning …. Like I never have any control of my life.” “I could not move; even picking up a cup required a serious attempt.”
Social/ intersubjective	“I am afraid of having relations with others, but I was not like this before.” “Part of what people say is upsetting, so I stay away from them.” “I miss the interdependence in marriage and at work; when you lose that, everything falls apart.”
Cognitive/ psychological	“It’s like a funky fogginess …. I cannot think, I cannot concentrate. My words end up not even coming out the way that they should.” “Just hundreds of thoughts whirling around in my head, with not function or order. It’s complete chaos.” “I was losing …. any sense of who I was.”
Reflective	“Every decision was segmented into a thousand tiny decisions. It came with a loss of being fully engaged in the world around you.” “The thoughts just come …. Sometimes I do not want to think but the thoughts just come. I try to stop them, but I cannot.” “I’m trying to change the subject, but my brain is telling me to worry about this, worry about that, and the next thing, I could not concentrate on anything else except what was in my head.”
Ecological	“I felt like in an artificial world that I did not recognize.” “I feel completely cut off from the rest of humanity, the rest of the world, the rest of existence.”
Normative	“I felt like my life was changed upside-down … I had become still and then driven down. I felt like nothing was important.” “I feel like what people talk about is trivial and irrelevant.” “Anyway, I felt that I must die … Everything would be over if I died. There would be no memory, painful memory, and no more real-world pressure. I felt that death could solve any problem.” “Public stigma is internalized into the self-stigma … that we are lazy, worthless.”

One can find numerous examples of how the dynamical processes of the self-pattern are intertwined. For example, withdrawal of intersubjective connections, as in the case of solitary confinement can lead to cognitive, affective, and motor difficulties (Gallagher, 2024). Solitary confinement can be a particularly devastating experience in that it degrades every factor in the person’s self-pattern beginning with the intersubjective dimension. Although similar difficulties can be found in ASD, a detailed contrast shows clear differences in the dynamics of behavior and appropriate therapies leading to improved social interactions (Gallagher, 2024, Ch. 10). Specifically, with regard to the narrative aspect of the self-pattern we can clearly see how narratives may play a dynamic role in configuring and regulating various aspects of the self; for example, in interacting with embodied and intersubjective processes in body dysmorphia and social anxiety. The *ESP* thus makes potentially fruitful links with the large body of work on master narrative theory (McLean et al., 2020) and intervention strategies developed in narrative therapy (Gallagher, 2023).

## 5 Self-selecting salience within a co-constructed phenomenological interview

The *ESP* provides a framework for mapping and tracking the patient’s experience which allows for a dynamic unfolding that is distinctively *not* aimed at establishing a classification as such, although a classification may be suggested in the constellation or pattern of expressed experiences. We propose that it is possible to enhance the openness of the interview, and at the same time both retrieve information suitable for conducting rigorous research as well as offer insights for skillful clinical practice, without the need for a fixed psychopathological classification.

One criticism of structured interviews is that they corral the patient’s experience into categories already defined and predetermined by some standard theoretical framework. In this way, the imposition of a fixed structure on the interview obscures what may be most salient for the interviewee/patient (the expert by experience) and may potentially elide relevant aspects. Such an interview process can impede richer understanding of the patient’s experience. This is why we emphasize the notion of self-selecting salience within the framework of a semi-structured interview. In the initial phase, the questions are very open, identifying a general area of relevance, while the direction remains focussed on whatever is identified by the patient as being most salient to them and the problem(s) they wish to explore and address at that particular time. If a conspicuously relevant issue is not mentioned, this does not mean that the issue is unimportant, but rather it may reflect the particular path the interview has followed, the reluctance of the interviewee to address the issue at this time or their perception of the interests and competence of the interviewer. Thus, the patient directs the orientation and starting point of the series of conversations. In the next phase of the interview, the elements and processes of the self-pattern are examined in more detail according to whatever was revealed in the initial phase.^12^

Contrary to Nordgaard et al. (2013) and Petitmengin et al. (2019), we propose that there is no need to aim for an absolutely faithful and meticulous recreation of the patient’s subjective experience. In fact, this may not even be possible given that trying to capture any experience is perforce retrospective.^13^ The narrative that emerges in such interviews may also be “prospective” insofar as it is a co-construction and this may be employed and validated by the subsequent interactions of the patient-clinician dyad or by others. Moreover, due to the interactive dyadic nature of any interview, ‘contamination’ of the testimony by the interviewer is impossible to eliminate entirely (despite, for example, the subtle refinements offered in the micro-phenomenological interview). Nor is such an aim necessarily therapeutically beneficial [see Hutto and Gallagher (2017)]. We stress that removing the requirement of an absolutely faithful re-creation need not be problematic. What is relevant is the understanding in the present moment which is supported by the ‘co-presence’ of both interviewer and interviewee.^14^ The interviewer is not merely a means for recording the interviewee’s experience, nor a passive observer of objectified contents but a participant in the conversational co-construction of a narrative self-presentation and self-understanding. Hence, we advocate a relational mode of interviewing that facilitates disclosure and understanding through a process of intersubjective meaning-making. The self-selecting salience is the starting point as explained above so that the interview maintains respect for what the ‘expert-by-experience’ sees as important and is also ready to discuss. The interview is nonetheless co-constructed, being two people engaged in dialogue (the patient and the clinician). While epistemic responsibility and authority are strongly accorded to the interviewee, the interview protocol we seek to implement nonetheless recognizes the dialogical and participatory roles of both interviewee and interviewer in articulating the unfolding narrative and negotiating a way forward [see, e.g., Kirmayer (2000)].

## 6 Frontloading the self-pattern in phenomenological interviews

There is a growing literature on the use of phenomenological interviews in psychiatric contexts. The phenomenological interview is a method generally used in qualitative research. It functions as a set of second-person interview questions and techniques, generating a narrative that in the psychiatric context can then be analyzed and interpreted for purposes of research (Høffding and Martiny, 2016; Køster and Fernandez, 2019, 2021; Gallagher and Zahavi, 2021; Frohn and Martiny, 2023). As mentioned above, variations of it can be found in the ‘Examination of Anomalous Self Experience’ (*EASE*) protocol (Parnas et al., 2005) and the more recent ‘Examination of Anomalous World Experience’ (*EAWE*) (Sass et al., 2017) and ‘Examination of Anomalous Fantasy and Imagination’ (EAFI) (Rasmussen et al., 2018). The strategy that we propose here for the *ESP* follows suggestions by Gallagher and Zahavi (2021) and Køster and Fernandez (2019, 2021), based on the concept of front-loading phenomenology.

Front-loaded phenomenology is an approach developed in the context of empirical research in cognitive science (Gallagher, 2003). In that context the approach involves using insights, concepts, and analyses found in phenomenological analyses to influence experimental design. Using this approach means that experimental subjects do not have to be trained in phenomenological methods. Gallagher and Zahavi (2021, p. 44) have suggested that frontloading phenomenological concepts can also shape the way that interviews are conducted. This means that, although interviewers will need to understand the concepts and methods of phenomenology, interviewees do not have the same requirement [see Bockelman-Morrow et al. (2013)].

Køster and Fernandez (2021), for example, suggest that interviews may frontload “what phenomenologists call ‘invariant existentials’ or ‘existential’ structures to provide a framework allowing the qualitative researcher to focus on a specific feature of human existence and investigate its particular modes.” In a similar fashion, Frohn and Martiny (2023) frontload the interview process with a framework defined by four enactive-phenomenological dimensions: the existential, biological, social, and psychological (following De Haan, 2020). Frontloading phenomenological existential or enactive dimensions means simply that the interview is guided by these elements. In other words, the open-ended questions of the phenomenological interview process, and the follow-up analysis are organized around these themes. In this way, it is possible to obtain information about each of the domains explored and the observation that the patient has little to say about particular domains itself may be noteworthy.

We have been developing a similar strategy, frontloading the elements of the self-pattern to structure the interview process, and analyzing the resulting narratives within that same framework. Frontloading the factors that make up the self-pattern, we suggest, provides both a more focused and specific interview (related to the self) and a more comprehensive set of data (in the sense of the number and range of factors explored) than that provided by existing phenomenological interviews. Although there is clearly much overlap with embodied, social, psychological and existential factors (which may include both affective and normative dimensions), organizing the interview around the self-pattern allows the interviewer to uncover the dynamic relationships between multiple processes of self-construction and construal.^15^ By emphasizing the dynamical relations among the processes of the self-pattern, for example, we can specify how affective processes in a particular patient directly relate to how they perceive the world and other people. Thus, if a patient is asked to explore the affective dimension of the self-pattern with questions from the interview —for example: “Can you describe how your mood has been recently?” — a patient may report “I experienced that I became more and more apathetic toward my own life… Even the things that I thought were fun normally, and which gave me something normally - it was all devoid of meaning” (cited in Frohn and Martiny, 2023, cf. Table 1). One can see in this response the connection between affectivity and ecological factors (affordances), as well as a possible modulation in the prereflective sense of agency – issues that can be explored in follow-up questions. Prompted by a question about how they feel when they are around other people, a patient may report fatigue and loneliness, suggesting dynamical relations between embodied and social processes in the self-pattern. In this way, the phenomenological interview can not only guide an idiographic analysis of individual experience, but when applied to a larger sample can also contribute to work in symptom network theory that aims to develop dynamical systems theories of mental disorders (Scheffer et al., 2024).

One might also find a different relation between a person’s sense of agency and their intersubjective relations. Responding to a question that explores intersubjective relations, one response might be: “I did not think I could control people’s thoughts, but I did often feel like I could influence how they felt and how … I do not think I could read people’s thoughts, but I could influence them, and that I could influence how they felt about things” (unpublished data from a patient diagnosed with schizophrenia).^16^ Whereas some patients with schizophrenia have problems with body boundaries, and feel invaded by others, this patient denies any confusion about boundaries. In this respect, the phenomenological interview can be very specific, delineating different experiences within a particular situation, in ways that reflect changes in the self-pattern, and sometimes in ways that resist the easy classifications of psychopathology. That is, interviews framed by factors of the self-pattern are less guided by checklists reflecting general psychiatric diagnoses, and more oriented toward interpretations that are patient specific.

## 7 Using the *ESP* framework in mental health research: methodological considerations for data collection and analysis

Given the flexibility of the proposed framework, researchers who choose to frontload the *ESP* into their research design may use different methods for data collection and data analysis. Here we focus more specifically on the use of the *ESP* framework within a qualitative paradigm, although the same framework can equally be used for the collection of quantitative data. While the *ESP* framework employs the phenomenological interview for data collection and encourages self-selecting salience and co-construction of meaning, as detailed above, there may be considerable variation in how the interview is conducted to reflect the context of interviewing, interviewee characteristics, or to address specific research questions.

Phenomenological interviews can vary in several regards: for example, their structure, such as where the conversation starts and ends, whether it covers predefined topics or proceeds in a minimally structured way, and whether it explores contextual information or focuses solely on pre-reflective aspects of experience. In addition, interviews may differ in relation to their setting, interactional dynamics, situational responsiveness, and discursive dimensions (Gubrium et al., 2012). All of these aspects, and many others, will affect the kind of narratives that will eventually be collected. Although researchers only have partial control over this process, especially given that interactional dynamics may not be fully accounted for in advance, we suggest that a life story interview approach [e.g., Atkinson (2012)] may be beneficial to initiate the conversation and facilitate the sensitive collection of personal narratives.^17^ To this end, participants, may be invited to create (or help the interviewer create) a lifeline drawing as a tool to guide them through the narration of their story (de Vries et al., 2017; Josselson and Hammack, 2021). The self-narrative obtained in this way may provide a window into different, interacting elements of the pattern that can help guide the rest of the interview.

The analysis of the co-constructed narratives can be achieved according to various methods. With qualitative research in general, methods will depend on the specific research problem(s) or question(s) to be addressed, study participants or data sources, prior understanding of the phenomena under study, overall study design, sample size, richness of the data, and so on. Methods and procedures used for analysis should be well-grounded in the objective of the study and should follow a principle of transparency.

Given the flexibility of the *ESP*, approaches drawing on thematic analysis (Braun and Clarke, 2022) may be considered when researchers are interested in capturing patterns of meaning across qualitative datasets. Thematic analysis includes a variety of different approaches within qualitative research and is not tied to any particular theoretical framework, and therefore would be suitable for the process of frontloading the *PTS* as outlined above. While thematic analysis involves a heterogeneous set of methods that can be conducted in different ways (e.g., using a deductive or inductive approach, an experiential or critical orientation, a realist or constructionist perspective), it usually involves a “categorizing”^18^ strategy such as coding.

In his argument for the value of applying a realist philosophical perspective, Maxwell (2012) cautions about the limitations of coding. Typically, coding proceeds through an initial phase of decontextualization and segmentation of the text into discrete “meaning units” that are labeled according to their content and then later grouped into “themes.” Themes may be inductively or deductively generated in various ways and combinations. In all cases, the process of theme generation relies on analysis of meaning patterns identified within the text in virtue of their contiguity and context, followed by the grouping in terms of patterns based on similarity (Maxwell, 2012). This process has implications for the exploration of a self-pattern understood as a dynamical gestalt, insofar as the overall changing shape of the pattern is determined by the existing, dynamical relations between its elements. For this reason, when interpreting the data for qualitative analysis, it is important that the unique relations of meaning between the elements are retained as much as possible, and that the data are interpreted with sensitivity to the particular embodied, experiential, and situated context in which they originated.

To avoid premature categorization into the different elements, potentially losing the meaning related to the dynamic links between elements in data analysis, we suggest a method in which the self-narrative interview transcripts are re-read several times in their entirety following the story line as drawn and narrated by participants.^19^ During these initial readings, the text may be annotated for anything significant that stands out, and for the relationship of elements to the overarching interview trajectory, in addition to any specific research questions. Josselson and Hammack (2021, pp. 28–29) provide a helpful list of markers of salience that can help identify what is potentially significant in the text. General first impressions may be annotated, and researchers may create a narrative summary or “profile” (Seidman, 2006) to hold on to the context of the interviews throughout the analytic processes. For instance, in research focused on psychopathological phenomena, this first analytic stage, using a connecting strategy, preserves the contiguity-based connection between events, people, emotions, memories and experiences narrated. After this contextual information is crafted and summarized, researchers may decide to take either a “categorizing” step (e.g., by looking at different elements of the pattern) and begin coding the text, or they may take a further “connecting” step (e.g., by looking for contiguity-based connections between elements).

While thematic analysis can be highly contextual in how codes are grouped into themes, it is not inherently so. Failure to recognize these issues may lead phenomenological researchers to “extract” a decontextualized utterance from the text in a way that risks distorting participants’ experiences to justify and support, for instance, psychopathological claims or theories (Stanier, 2022). This may increase the risks of epistemic harms such as those described by Spencer and Broome in relation to certain contemporary forms of empathic understanding in psychiatric research and include risks of error “leading to an overall misunderstanding of the experience at hand, and epistemic injustice through co-opting the patient’s experience and intellectual arrogance and epistemic objectification” (Spencer and Broome, 2023).

To mitigate these risks during the analytic process, we propose using a combination of coding and “connecting” strategies iteratively when analyzing *ESP*-frontloaded interview data. Connecting strategies emphasize the preservation of data in their original form and rely on analytic techniques aimed at identifying contiguity-based relations of meaning embedded into a self-narrative. This analytic move is central to exploring self-patterns insofar as this process may unveil the key relationships that tie the elements together into a dynamical gestalt, and give rise to specific experiential, psychological, behavioral, intersubjective manifestations. Consider, for example, the patient’s response cited earlier: “I experienced that I became more and more apathetic toward my own life… Even the things that I thought were fun normally, and which gave me something normally - it was all devoid of meaning” (cited in Frohn and Martiny, 2023, cf. Table 1). To categorize this as reflecting a modulation of affectivity is not wrong, but one also needs to mark out the connection between affectivity and the diminishment of affordances (ecological factors), as well as what seems to be a diminishment in the prereflective sense of agency. Analysis needs to understand the connections made in this part of the narrative and how it may connect with what is expressed in the full narrative. There are several qualitative approaches that emphasize connecting strategies in the process of data analysis, including different forms of narrative inquiry (Lieblich et al., 1998; Crossley, 2000; Riessman, 2008; Josselson and Hammack, 2021).

It is also important to recognize that a form of narrative analysis can occur throughout the interview process rather than separately after data collection, in an approach that presupposes the co-construction of meaning between researcher and participant. Specifically, this ongoing analysis is implicit in selection of follow-up questions by the interviewer, based on the interviewee’s responses, exploring, for example, the connections between affect and sense of agency. This will depend on the analytic skills of the interviewer to see the connections that need to be explored further. These decisions about what connections to explore during the interview, either by interviewee or interviewer, are also things that need to be considered in subsequent interpretive analysis of the narrative.

As Maxwell (2012) has suggested, rather than viewing these strategies as alternating or sequential, it may be more helpful to view them as connecting and coding “moves” Maxwell (2012, p. 119). Combining these approaches in a complementary manner is likely to yield a more in-depth, richer, and pluralistic understanding of particular phenomena, whether psychopathological or otherwise. This approach may incorporate pre-reflective, embodied, subjective, behavioral, reflective, narrative and intersubjective processes within a given personal, ecological, and normative context.

While the above focuses on qualitative methods, there is much potential for the *ESP* framework to be used in mixed-methods or quantitative settings, which may require varying levels of modification to what has been suggested above. To give a few examples: data from the *ESP* framework could explore via various qual-quant coding approaches; the characteristics and content of speech could be examined, and/or patterns from the *ESP* examined in any variety of quantitative approaches (frequency of terms, coherence of speech, acoustic signatures of speech, frequency of themes, formalized coding and scoring of responses). The format of the *ESP* interview needn’t be completely reconfigured, however, as both content and speech from the interview could be subject to acoustic analysis and natural language processing to ascertain patterns in the data [see, e.g., Kishimoto et al. (2022)]. Patterns extracted from the *ESP* could also be considered within or compared against more traditional psychodiagnostics assessment tools to further develop understanding of mental illness relative to current mainstream approaches.

## 8 Potential controversies and limitations

The use of the self-pattern as outlined above to guide an interview approach addresses a concern raised by Russell (2023) about enactive approaches to psychiatry, such as those proposed by De Haan (2020), Maiese (2022), and Nielsen (2020).

For enactive psychiatry to ‘do work’ in the domain of mental illness, it should not just confirm the hypotheses of psychiatry already which may have drawn its conclusions as to what is pathological on the basis of models of disorder that enactivism has criticized (such as brain-based models). In other words, enactivism cannot presuppose pathological behavior and then reconceptualize it according to its own framework as this will give the framework a false sense of success; in order to be more ontologically sound and make strong empirical claims, enactivism should be able to point toward disordered behaviors without appealing to prior presuppositions about which behaviors are pathological (Russell, 2023, p. 1472).

Of course, for many practical reasons, including institutional constraints (social, economic, and political) on the health care system, clinicians do not have the option of entirely giving up the presuppositions associated with current psychiatric classifications. Methodologically, however, to the extent that enactive approaches, or approaches that focus on the self-pattern can put phenomenological interview techniques to use, psychiatric classifications need not be the sole or determining factor in understanding a patient’s experience, or in therapeutic practice. Køster and Fernandez address a similar point in response to the worry that frontloaded categories might seem to bias the outcome. As they argue:

Front-loading the interview does not predetermine the content of the interviewees’ descriptions. There is a significant difference between predefining the focus of an investigation and predefining what will emerge from this focus. That is, a predefined focus on existential feelings does not dictate the kind of alteration that might emerge (Køster and Fernandez, 2021, p. 161).

Likewise, framing the interview in terms of the self-pattern facilitates an open, explorative perspective that focuses on the patient’s experience across a comprehensive set of factors that have specific import for their everyday life, rather than on a predefined set of syndromes (Fried, 2022). In addition, the patient and clinician co-direct the course of the interview, ensuring coverage of factors relevant to the PTS (see Table 1) but allowing the attention given to specific factors to be guided by their salience for the patient.

In response to Russell’s objection one can also point out that the narratives generated in phenomenological interviews often uncover self-pattern processes that standard checklist models, like the *DSM*, fail to note [see, Gallagher and Daly (2018), Daly and Gallagher (2019), and Fusar-Poli et al. (2023)]. In this regard, Frohn and Martiny (2023) point out that phenomenological interviews are able to discriminate between what they call “patho-description” and more authentic reports. Patho-descriptions include both those descriptions that tend to be the result of the patient’s familiarity with their diagnosis, and those descriptions that are the result of the psychopathology itself. These may include narratives that are compensatory or defensive, or that build on processes of the self-pattern that may be protective or adaptive, even if entangled with disordered components (Stanghellini et al., 2022b). With respect to depression, for example, Frohn and Martiny explain:

If we start by looking at the descriptive style and narratives that the participants use when they describe their experiences, we see examples where depression influences how they access and describe their own experiences. This is possible to see with the help of the phenomenological interview, since its specific methodology helps the participants in some instances to describe their experiences in more detailed and nuanced ways. This means that while the participants do retell and reproduce the same general, negative stories about depression, which corroborate the current phenomenological model of depression, they also provide new descriptions that differentiate from - and seem to conflict with - some of these general, negative stories (Frohn and Martiny, 2023, Section 5.1).

Descriptions of negative symptoms and a lack of a sense of agency, consistent with the standard views of depression, are mixed with reports of circumstances in which there may be an increased sense of agency and seemingly contradictory “different feelings such as anger, fun, and bodily pain,” picking up on experiences that are underplayed in the *DSM-5* which primarily focuses on the negative aspects of the depressive mood. On the self-pattern view, such variations can be explained not as an abstract set of symptoms, or simply by a difference between prereflective and reflective processes (as Frohn and Martiny, 2023 suggest), but as the result of the overall dynamical relations among factors in the self-pattern that, given specific circumstances, are characterized by different weights, e.g., when specific social circumstances take on a high significance, in contrast to being alone, or when in a specifically supportive environment one’s bodily affect is attuned to a task. In this regard, what we call a “disorder” is not conceived on the abnormal view of psychopathology, either as deviations from normal biological or psychological functioning (Matthewson and Griffiths, 2017) or as purely mental dysfunctions located within the individual’s head. It is rather a characterization of the self-pattern (situated in an environment) that may be disrupted or that may be the result of the agent’s coping (successfully or unsuccessfully) with anomalous experiences.

Russell (2023) rightly raises a related issue that concerns hermeneutical justice. A patient’s experience of psychopathology are shaped by cultural preconceptions of what that experience is like, which in turn is closely tied to diagnostic criteria and what it means to be a person with particular anomalous experiences. The patient thus starts to define themselves and the limits of their possibilities in terms of the disorder (Haslam, 2016). As Russell writes: “This is tantamount to hermeneutical and testimonial injustice … injustices as a result of harms suffered by the testimony giver due to (a) inaccessibility to certain concepts to make sense of their experience (hermeneutical injustice) and/or (b) the individual being ascribed less credibility due to some feature of them as a person (testimonial injustice)” (Russell, 2023, p. 1473; see also Fricker, 2007 and Haslanger, 2019). In an interview setting, even if a person is able to report that her own experience is pathological, she will be describing a view that is caught up in a mix of empirical and normative claims (Alexandrova, 2018). While this may result in injustice when there is a marked power differential and the patient is constrained in articulating her own perspective, it is important to acknowledge that all conversations whether clinical or otherwise involve mutual constraints. The *ESP* thus begins with a minimally structured interview so as to avoid the structuring effects of diagnostic labeling. This approach is similar to the way these concerns were addressed in the format of the *McGill Illness Narrative Interview* (Groleau et al., 2006).^20^

For this reason, when using the *ESP* an attitude of *hermeneutical flexibility* is encouraged to mitigate the risk of hermeneutical injustice (Ritunnano, 2022). In each case interviewers (whether clinicians or researchers) will find themselves situated in the context of an epistemic interaction where the interviewee—in relating different facets of their experience of mental disorder—is creating a self-narrative. This self-narrative, in addition to providing a window into the complex and dynamic alterations of the self-pattern, is also affected by the very encounter in which the patient finds herself. In other words, in the interview, both telling and listening are shaped by several different factors such as our social identities, that is, who we are as members of a social group (e.g., our ethnicity, gender, religion, age, socio-economic status, etc.); the context and purpose of that particular interaction (whether, for instance, this is responding to a specific need of the individual who is seeking a diagnosis or otherwise has been set up by the researcher with a specific agenda in mind); the presence, perception and attitude toward the interviewer (both their personal characteristics and social position); and the range and availability of hermeneutical resources that are available to both interviewer and interviewee to draw upon in their mutual process of self-interpretation and meaning co-construction.

For instance, in the case of an interview conducted between a mental health professional and a patient, who we are and what power we have matters. Patients may not want to tell their story in the first place because, for instance, they have had previous negative experiences of mental health services and have lost trust, or because they may worry about the repercussions of their story (e.g., being ‘labeled’ or involuntarily admitted to hospital), or they may be struggling to express their experience and make it intelligible to others because there are simply no words available to describe it. Similarly, in the context of interviewing, factors such as settings, the professional background and personal characteristics of the researcher and the respondent are equally important and may influence interpretation and the generation of meanings at different stages of the research - from data collection to data analysis and writing-up.

For all these reasons, it is recommended that researchers and clinicians alike cultivate an attitude of hermeneutical and discursive flexibility where conversations and ensuing narratives are not seen as objects but as dynamic acts of sense-making with inherent ambiguities and contradictions [Ritunnano, 2022; Russell, 2023; Spencer, 2023; for an example applied to the attribution of psychotic symptoms with religious experience, see Porcher (2023)].

## 9 Concluding thoughts

In this article, we have argued that the use of phenomenological interviewing in the context of psychopathology and mental health more broadly, can benefit from a conceptual grounding in the pattern theory of self. The *PTS* foregrounds the multi-dimensionality of the subject, stressing not only situated embodiment but also the significance of intersubjective, ecological, and normative processes in the formation of self-patterns. We have discussed the evolution of understandings that have contributed to the development of a novel phenomenological interview framework, which we have referred to as the *Examination of Self Patterns* (*ESP*). On this approach, the elements of the self-pattern provide a frontloaded framework that can guide the interview process, thereby informing the development of an interview protocol. This strategy, moreover, enables the co-construction of meaningful narratives through processes emphasising self-selecting salience, ensuring that the patient/ interviewee retains a significant measure of epistemic responsibility and authority. In this way, the *ESP* can facilitate the discovery of previously occluded narratives and reveal hidden relations of meaning through a dynamical mapping of different elements of the self.

The same framework, therefore, can be used to guide the analysis of the resulting narratives in a way that is alert to risks of epistemic and hermeneutical forms of injustice in clinical and research settings. We have suggested that a combination of connecting and coding analytic strategies may help mitigate these risks during the analytic process in qualitative studies. A more direct form of validation could involve discussing the interpretations with the patient themselves to evaluate whether the interpretations are consistent with their experience and/or offer insight and potentially useful directions for addressing the problems [see, e.g., Hawke et al. (2022)]. Responses could also be compared to clinician impressions and/or observations from the interview process. This approach and the use of multiple methods for triangulation can increase the trustworthiness and validity of interview data.

The *PTS* and *ESP* align with a broader renewal movement, within phenomenological psychopathology, that acknowledges the importance of relational, normative, and ecological contexts in the shaping of the self across a variety of mental health challenges. The value and versatility of the *PTS* approach have been amply demonstrated by its wide application across various disciplines within the applied human sciences, extending beyond the domain of psychopathology. These applications span numerous topics, such as discussions of deep brain stimulation, *AI*, Buddhist psychology, mind–body analyses, technology studies, rehabilitation studies, and psychedelic therapy among others. We strongly encourage further empirical applications to advance the development of the *ESP*, involving a diverse array of voices and stakeholders in both research and clinical settings. The *ESP Interview Protocol* is currently being finalized and will made available in a later publication.

## Data availability statement

The original contributions presented in the study are included in the article/supplementary material, further inquiries can be directed to the corresponding author/s.

## Ethics statement

Ethical approval was not required for the study involving humans in accordance with the local legislation and institutional requirements. Written informed consent to participate in this study was not required from the participants or the participants’ legal guardians/next of kin in accordance with the national legislation and the institutional requirements.

## Author contributions

AD: Conceptualization, Investigation, Methodology, Writing – original draft, Writing – review & editing. RR: Conceptualization, Investigation, Methodology, Writing – original draft, Writing – review & editing. SG: Conceptualization, Investigation, Methodology, Writing original draft, Writing - review & editing. LK: Conceptualization, Investigation, Methodology, Writing - original draft, Writing - review & editing. NVD: Conceptualization, Investigation, Methodology, Writing - original draft, Writing - review & editing. JK: Investigation, Methodology, Writing - review & editing.

## Footnotes

^1^For more extended critiques of RDoC, see Kirmayer and Crafa (2014), Paris and Kirmayer (2016) and Gómez-Carrillo et al. (2023).

^2^Throughout the paper, we refer to “mental disorders” as well as to the idea of a “disordered” self-pattern. Within psychiatry, psychology and philosophy of mind, the question of “what counts as a disease or disorder” is highly disputed (Wakefield, 2007; Cooper, 2020). Our proposed *ESP* framework does not settle this long-running debate. However, while we do not directly address questions in this paper concerning the nature and classifications of disorders in mental health, we agree with Bortolotti (2020) that this need not be a problem—neither for the status of psychiatry and medicine in general, nor for the specific aims of our project. Progress can be made, and insight gained (even without a well-defined demarcation criterion) if we accept that people may experience a variety of medical and psychiatric problems leading to suffering and disability, and they may seek help irrespective of whether we classify such problems and experiences as pathological. Similarly, our aim is not to come up with necessary and sufficient conditions for a given configuration of the self-pattern to be classified as disordered; making such distinctions is not the aim of the *Examination of Self-Patterns* (*ESP*). However, we acknowledge that, in some circumstances, some configurations may lead to suffering or problems in living that may or may not reflect a dysfunction in a biological or diagnostic sense.

^3^Several studies have indicated the viability of the *PTS* as a basis for understanding mental disorders (Dings and de Bruin, 2016; Fingelkurts and Fingelkurts, 2017; Baird, 2019; Fingelkurts et al., 2020, 2021; Neustadter et al., 2021; Ciaunica et al., 2022: Dings and de Bruin, 2022; Zawadzki, 2022; Giommi et al., 2023), or more broadly for self-understanding (Newen, 2018; Lindahl and Britton, 2019; Motta, 2023).

^4^In short, we note that a change in the self-pattern becomes a disturbance when that pattern becomes stuck in a form that contributes to maladaptive function [in dynamic systems theory, this is consistent with the idea of an attractor or valley that corresponds to a “resilient disorder state” – see, e.g., Scheffer et al. (2024)]. Furthermore, studying the dynamic relationships between different aspects of the self-pattern is a potential fruitful line of future research.

^5^For clarification of the role of phenomenology in qualitative research see, Zahavi (2020). On “objectivity” and the epistemic role of the patient see Tekin’s concept of *Participatory Interactive Objectivity* (Tekin, 2022); on participatory research practices, and standpoint theory see Friesen and Goldstein (2022).

^6^For example, the data generated in an *ESP* study additionally could be examined using *Natural Language Processing* (Kishimoto et al. 2022) and/or other approaches to examining the features of speech (e.g., acoustic tone, temporal dynamics of speech). Interviewer observations of mental state and behavior during the interview are additional sources of information. Such data could be used on its own, or in conjunction with data obtrained from other apsects of the framework and diagnostic process towards a more thorough 1st, 2nd, and 3rd person understanding of psychopathology.

^7^In particular, narratives may map facets of the self that link past history (memory) with future (possible) selves. See Bouizegarene et al. (2024).

^8^And this is not to say that it will always capture all the aspects of the self-pattern at the one time but may favor certain aspects and possibly occlude or contradict other aspects. Nonetheless, the narrative that the interviewee is communicating at that time is the one that is salient and communicable.

^9^In many cases, the self may be expressed through metaphors rather than more extended narratives, see, Kirmayer (2023).

^10^For a global survey of psychiatrists’ attitudes to mental disorders classifications, see Reed et al. (2011).

^11^For a related paper which develops the idea of a pattern theory of scaffolding, with regard to mathematical cognition with implications for research into embodied, embedded, extended, and enactive (4E) cognition see Newen and Fabry (2023).

^12^The specific phases and strategies of the interview will be addressed in full detail in the next paper setting out the ESP protocol.

^13^Merleau-Ponty (2012, pp.361 & 362) anticipates this difficulty when he writes: “… and I can never be certain of understanding my past better than it understood itself while I lived it ... tomorrow, with more maturity and more insight, I will perhaps understand my past differently and I will accordingly construct it differently.”

^14^See also Daly and McCaw (2024).

^15^Køster and Fernandez (2021, p. 165), for example, state that their approach, which they term *Phenomenologically Grounded Qualitative Research* is focused on the prereflective level of experience and involves studies that “investigate broad existential orientations and characterize alterations in the overall mode of being in the world of the person, partitioned through the lens of existentials”. The self-pattern includes much more than prereflective experiences.

^16^Ritunnano (2022), data from the MELBA study, manuscript in preparation, IRAS Project ID 317572, ERN_2022–0325.

^17^There are of course other strategies that may work just as well when simple open-ended questions are insufficient to initiate the conversation. The clinician/researcher will likely have a repertoire of strategies that they can draw on and will recognize which strategy will be most effective for the specific situation and the specific patient.

^18^“Categorizing” here does not denote a reductive method of diagnostic classification as found in the *DSM*, but rather refers to Maxwell’s (2012) methodology in which “categorizing” involves the analytic strategies of coding in qualitative analysis. In the process of coding, qualitative data are segmented into discrete units of text, which are then labeled and grouped into categories as a way of sorting the descriptive data into a similarity-based ordering that replaces the original contiguity-based relationships found in the text.

^19^This raises an interesting and important issue: locating self-experience within a life story will necessarily highlight aspects of the self that fit with the narrative self and with the long-arc of biography and the vagaries of autobiographical memory; in contrast, for example, a focus on ‘turning points’, or symptom onset, coping or resolution will favor other aspects of the self (perhaps those that are more explicitly embodied -- depending on the nature of the symptoms) this effect of the overall framing of the interview will influence both the ways that people talk about their experience and, if it is used as a frame for initial analysis, the ways in which the interviewer interprets the data [see Corin and Lauzon (1992), Corin et al. (2005), and Windell et al. (2015)].

^20^The MINI (McGill Illness Narrative Interview) anchors the interview in particular symptoms, problems, or experiences that are salient to the patient. It begins by asking the patient what happened around the time specific symptoms or experiences first occurred. This elicits an initial ‘chain complex’ narrative ordered by contiguity. After that has been elaborated, the patient is asked if there are any prototypes for the experience either in their own previous experience (Have you ever had anything like this before?) or that of others around them or in other sources (e.g., media). Finally, the patient is asked explicitly about their own understanding of the nature of the symptoms or problem in terms of labels, causal attributions and explanatory models. By moving from open to more specific forms of narration, the interview gives the patient the opportunity to describe symptoms in their own terms rather than through generic accounts drawn from conventional cultural models and categories. Although in practice the chain complex, prototypes and explanatory model narrative strands are often intertwined, they can be distinguished in illness narrative interview transcripts (Stern and Kirmayer, 2004).

## References

[ref1] Alexandrova A. (2018). Can the science of wellbeing be objective? British J. Phil. Sci. 69, 421–445. doi: 10.1093/bjps/axw027

[ref2] Andreasen N. C. (2007). DSM and the death of phenomenology in America: an example of unintended consequences. Schizophr. Bull. 33, 108–112. doi: 10.1093/schbul/sbl054 17158191 PMC2632284

[ref3] Atkinson R. (2012). “The life story interview as a mutually equitable relationship” in The SAGE handbook of interview research: The complexity of the craft. Eds. Gubrium, J. F., Holstein, J. A., Marvasti, A. B., & McKinney, K. D. SAGE Publications, Inc. doi: 10.4135/9781452218403

[ref4] Baird A. (2019). A reflection on the complexity of the self in severe dementia. Cogent Psychol. 6:1574055. doi: 10.1080/23311908.2019.1574055

[ref5] Bieling P. J. Kuyken W. (2003). Is cognitive case formulation science or science fiction? Clin. Psychol. Sci. Pract. 10, 52–69. doi: 10.1093/clipsy.10.1.52

[ref6] Bockelman-Morrow P. Reinerman L. Gallagher S. (2013). Methodological lessons in neurophenomenology: review of a baseline study and recommendations for research approaches. Front. Hum. Neurosci. 7:608. doi: 10.3389/fnhum.2013.00608, PMID: 24133430 PMC3794193

[ref7] Bortolotti L. (2020). Doctors without ‘disorders’. Aristotelian Soc. Suppl. Vol. XCIV 94, 163–184. doi: 10.1093/arisup/akaa006

[ref8] Bouizegarene N. Ramstead M. J. Constant A. Friston K. J. Kirmayer L. J. (2024). Narrative as active inference: an integrative account of cognitive and social functions in adaptation. Front. Psychol. 15, 1–19. doi: 10.3389/fpsyg.2024.1345480 PMC11188712 38903472

[ref9] Bracken P. Thomas P. Timimi S. Asen E. Behr G. Beuster C.. (2012). Psychiatry beyond the current paradigm. Br. J. Psychiatry 201, 430–434. doi: 10.1192/bjp.bp.112.109447 23209088

[ref10] Braun V. Clarke V. (2022). Thematic analysis: A practical guide. Thousand Oaks, California: SAGE Publications Ltd.

[ref11] Broome M. R. (2020). “The inhuman and human gaze in psychiatry, psychopathology and schizophrenia” in Perception and the inhuman gaze: Perspectives from philosophy, phenomenology and the sciences. eds. Daly A. Moran D. Cummins F. Jardine J. (New York: Routledge), 176–191.

[ref12] Broome M. R. Harland R. Owen G. S. Stringaris A. (2013). The Maudsley reader in phenomenological psychiatry. Cambridge: Cambridge University Press.

[ref13] Ciaunica A. Seth A. Limanowski J. Hesp C. Friston K. J. (2022). I overthink—therefore I am not: an active inference account of altered sense of self and agency in depersonalisation disorder. Conscious. Cogn. 101:103320. doi: 10.1016/j.concog.2022.103320 35490544 PMC9130736

[ref14] Cooper R. (2020). The concept of disorder revisited: robustly value-laden despite change. Proceed. Aristotelian Soci. Suppl. 94, 141–161. doi: 10.1093/arisup/akaa010

[ref15] Corin E. Lauzon G. (1992). Positive withdrawal and the quest for meaning: the reconstruction of experience among schizophrenics. Psychiatry 55, 266–278. doi: 10.1080/00332747.1992.11024600 1509013

[ref16] Corin E. Thara R. Padmavati R. (2005). Shadows of culture in psychosis in South India: a methodological exploration and illustration. Int. Rev. Psychiatry 17, 75–81. doi: 10.1080/09540260500050016 16194775

[ref17] Crossley M. (2000). Introducing narrative psychology: McGraw-Hill Education.

[ref18] Cuthbert B. N. (2022). Research domain criteria (RDoC): Progress and potential. Curr. Dir. Psychol. Sci. 31, 107–114. doi: 10.1177/09637214211051363 35692384 PMC9187047

[ref19] Daly A. Gallagher S. (2019). Towards a phenomenology of self-patterns in psychopathological diagnosis and therapy. Psychopathology 52, 33–49. doi: 10.1159/000499315, PMID: 31018215 PMC6878753

[ref20] Daly A. McCaw C. (2024). “Between phenomenology and mindfulness: the role of presence in the clinical and therapeutic context” in Routledge handbook of phenomenology of mindfulness. eds. Ferrarello S. Hadjiouannou C. (New York, London: Routledge), 353–366.

[ref21] De Haan S. (2020). Enactive psychiatry. Cambridge: Cambridge University Press.

[ref22] De Haan S. Rietveld E. Stokhof M. Denys D. (2017). Becoming more oneself? Changes in personality following DBS treatment for psychiatric disorders: experiences of OCD patients and general considerations. PLoS One 12:e0175748. doi: 10.1371/journal.pone.0175748 28426824 PMC5398533

[ref23] de Vries B. LeBlanc A. J. Frost D. M. Alston-Stepnitz E. Stephenson R. Woodyatt C. R. (2017). The relationship timeline: a method for the study of shared lived experiences in relational contexts. Adv. Life Course Res. 32, 55–64. doi: 10.1016/j.alcr.2016.07.002 28584522 PMC5454772

[ref24] Decker H. S. (2013). The making of DSM-III: A diagnostic manual's conquest of American psychiatry. Oxford: Oxford University Press.

[ref25] Dings R. de Bruin L. (2016). Situating the self: understanding the effects of deep brain stimulation. Phenomenol. Cogn. Sci. 15, 151–165. doi: 10.1007/s11097-015-9421-3

[ref26] Dings R. de Bruin L. (2022). What’s special about ‘not feeling like oneself’? A deflationary account of self(−illness) ambiguity. Philos. Explor. 25, 269–289. doi: 10.1080/13869795.2022.2051592

[ref27] Fingelkurts A. A. Fingelkurts A. A. (2017). Three-dimensional components of selfhood in treatment-naive patients with major depressive disorder: a resting-state qEEG imaging study. Neuropsychologia 99, 30–36. doi: 10.1016/j.neuropsychologia.2017.02.020, PMID: 28254650

[ref28] Fingelkurts A. A. Fingelkurts A. A. Kallio-Tamminen T. (2020). Selfhood triumvirate: from phenomenology to brain activity and back again. Conscious. Cogn. 86:103031. doi: 10.1016/j.concog.2020.103031, PMID: 33099083

[ref29] Fingelkurts A. A. Fingelkurts A. A. Kallio-Tamminen T. (2021). Self, me and I in the repertoire of spontaneously occurring altered states of selfhood: eight neurophenomenological case study reports. Cogn. Neurodyn. 16, 255–282. doi: 10.1007/s11571-021-09719-5 35401860 PMC8934794

[ref30] Fricker M. (2007). Epistemic injustice: Power and the ethics of knowing. Oxford: Oxford University Press.

[ref31] Fried E. I. (2022). Studying mental health problems as systems, not syndromes. Curr. Dir. Psychol. Sci. 31, 500–508. doi: 10.1177/09637214221114089

[ref32] Friesen P. Goldstein J. (2022). Standpoint theory and the Psy sciences: can marginalization and critical engagement Lead to an epistemic advantage? – corrigendum. Hypatia 38:450. doi: 10.1017/hyp.2023.45

[ref33] Frohn O. O. Martiny K. M. (2023). The phenomenological model of depression: from methodological challenges to clinical advancements. Front. Psychol. 14:1215388. doi: 10.3389/fpsyg.2023.1215388 38023023 PMC10658893

[ref34] Fulford K. W. Broome M. Stanghellini G. Thornton T. (2005). Looking with both eyes open: fact and value in psychiatric diagnosis? World Psychiatry 4, 78–86. 16633513 PMC1414736

[ref35] Fusar-Poli P. Estradé A. Stanghellini G. Esposito C. M. Rosfort R. Mancini M.. (2023). The lived experience of depression: a bottom-up review co-written by experts by experience and academics. World Psychiatry 22, 352–365. doi: 10.1002/wps.21111, PMID: 37713566 PMC10503922

[ref36] Galbusera L. Fellin L. (2014). The intersubjective endeavor of psychopathology research: methodological reflections on a second-person perspective approach. Front. Psychol. 5:1150. doi: 10.3389/fpsyg.2014.01150 25368589 PMC4201097

[ref37] Gallagher S. (2003). Phenomenology and experimental design: toward a phenomenologically enlightened experimental science. J. Conscious. Stud. 10, 85–99.

[ref38] Gallagher S. (2013). A pattern theory of self. Front. Hum. Neurosci. 7:443. doi: 10.3389/fnhum.2013.00443 23914173 PMC3730118

[ref39] Gallagher S. (2023). “Narrative as an interpretation of self-pattern” in Hanna Meretoja and Mark Freeman (eds.), The Use and Abuse of Stories: New Directions in Narrative Hermeneutics, Oxford: Oxford University Press. 136–153. doi: 10.1093/oso/9780197571026.003.0007

[ref40] Gallagher S. (2024). The self and its disorders. Oxford: Oxford University Press.

[ref41] Gallagher S. Daly A. (2018). Dynamical relations in the self-pattern. Front. Psychol. 9:664. doi: 10.3389/fpsyg.2018.00664 29867642 PMC5958307

[ref42] Gallagher S. Zahavi D. (2021). The phenomenological mind. London, New York: Routledge.

[ref43] Gilbert F. Viaña J. N. (2018). A personal narrative on living and dealing with psychiatric symptoms after DBS surgery. Narrat Inq Bioeth. 8, 67–77. doi: 10.1353/nib.2018.0024 29657181

[ref44] Giommi F. Bauer P. R. Berkovich-Ohana A. Barendregt H. Brown K. W. Gallagher S.. (2023). The (in) flexible self: psychopathology, mindfulness, and neuroscience. Int. J. Clin. Health Psychol. 23:100381. doi: 10.1016/j.ijchp.2023.100381, PMID: 36969914 PMC10033904

[ref45] Gómez-Carrillo A. Paquin V. Dumas G. Kirmayer L. J. (2023). Restoring the missing person to personalized medicine and precision psychiatry. Front. Neurosci. 17:1041433. doi: 10.3389/fnins.2023.1041433 36845417 PMC9947537

[ref46] Groleau D. Young A. Kirmayer L. J. (2006). The McGill illness narrative interview (MINI): an interview schedule to elicit meanings and modes of reasoning related to illness experience. Transcult. Psychiatry 43, 671–691. doi: 10.1177/1363461506070796 17166953

[ref47] Gubrium J. F. Holstein J. A. Marvasti A. B. McKinney K. D. (Eds.) (2012). The SAGE handbook of interview research: the complexity of the craft. Thousand Oaks, California: Sage Publications.

[ref48] Haslam N. (2016). Looping effects and the expanding concept of mental disorder. J. Psychopathol. 22, 4–9.

[ref49] Haslanger S. (2019). Cognition as a social skill. Australasian Phil. Rev. 3, 5–25. doi: 10.1080/24740500.2019.1705229

[ref50] Hawke L. D. Sheikhan N. Y. Jones N. Slade M. Soklaridis S. Wells S.. (2022). Embedding lived experience into mental health academic research organizations: critical reflections. Health Expect. 25, 2299–2305. doi: 10.1111/hex.13586 35999670 PMC9615091

[ref51] Heckers S. (2015). (2015). The value of psychiatric diagnoses. JAMA Psychiatry 72, 1165–1166. doi: 10.1001/jamapsychiatry.2015.2250 26560736

[ref52] Høffding S. Martiny K. (2016). Framing a phenomenological interview: what, why and how. Phenomenol. Cogn. Sci. 15, 539–564. doi: 10.1007/s11097-015-9433-z

[ref53] Hutto D. Gallagher S. (2017). Re-authoring narrative therapy: opening the way for future developments. Phil. Psychiatry Psychol. 24, 157–167. doi: 10.1353/ppp.2017.0020

[ref54] Hyman S. E. (2010). The diagnosis of mental disorders: the problem of reification. Annu. Rev. Clin. Psychol. 6, 155–179. doi: 10.1146/annurev.clinpsy.3.022806.091532 17716032

[ref55] Insel T. (2022). Healing: Our path from mental illness to mental health. New York: Random House.

[ref56] Jaspers K. (1913). Allgemeine psychopathologie: ein leitfaden für studierende, ärzte und psychologen. Heidelberg: Springer.

[ref57] Jaspers K. (1963) in General psychopathology. eds. Hoenig J. Hamilton M. W. (Manchester: Manchester University Press).

[ref58] Josselson R. Hammack P. L. (2021). Essentials of narrative analysis: American Psychological Association.

[ref59] Kendler K. S. (2016). (2016). The nature of psychiatric disorders. World Psychiatry 15, 5–12. doi: 10.1002/wps.20292 26833596 PMC4780286

[ref60] Kendler K. S. (2024). (2024). Are psychiatric disorders brain diseases? A new look at an old question. JAMA Psychiatry 81, 325–326. doi: 10.1001/jamapsychiatry.2024.0036, PMID: 38416478

[ref61] Kirmayer L. J. (2000). “Broken narratives: clinical encounters and the poetics of illness experience” in Narrative and the cultural construction of illness and healing. eds. Mattingly C. Garro L. (Berkeley: University of California Press), 153–180.

[ref62] Kirmayer L. J. (2015). “Re-visioning psychiatry: toward an ecology of mind in health and illness” in Re-visioning psychiatry: Cultural phenomenology, critical neuroscience, and global mental health. eds. Kirmayer L. J. Lemelson R. Cummings C. A. (New York: Cambridge University Press), 622–660.

[ref63] Kirmayer L. J. (2023). Cultural poetics of illness and healing. Transcult. Psychiatry 60, 753–769. doi: 10.1177/13634615231205544 37933139 PMC10744186

[ref64] Kirmayer L. J. Crafa D. (2014). What kind of science for psychiatry? Front. Hum. Neurosci. 8:435. doi: 10.3389/fnhum.2014.00435 25071499 PMC4092362

[ref65] Kirmayer L. J. Gomez-Carrillo A. Veissière S. (2017). Culture and depression in global mental health: an ecosocial approach to the phenomenology of psychiatric disorders. Soc. Sci. Med. 183, 163–168. doi: 10.1016/j.socscimed.2017.04.034, PMID: 28478959

[ref66] Kishimoto T. Nakamura H. Kano Y. Eguchi Y. Kitazawa M. Liang K. C.. (2022). Understanding psychiatric illness through natural language processing (UNDERPIN): rationale, design and methodology. Front. Psychol. 13. doi: 10.3389/fpsyt.2022.954703 PMC9752868 36532181

[ref67] Køster A. Fernandez A. V. (2019). On the subject matter of phenomenological psychopathology. In the Oxford handbook of phenomenological psychopathology. Oxford: Oxford University Press.

[ref68] Køster A. Fernandez A. V. (2021). Investigating modes of being in the world: an introduction to phenomenologically grounded qualitative research. Phenomenol. Cogn. Sci. 22, 149–169. doi: 10.1007/s11097-020-09723-w

[ref69] Kyselo M. (2014). The body social: an enactive approach to the self. Front. Psychol. 5:986. doi: 10.3389/fpsyg.2014.00986 25309471 PMC4162380

[ref70] Lieblich A. Tuval-Mashiach R. Zilber T. (1998). Narrative research: Reading, analysis, and interpretation. Thousand Oaks, California: Sage Publications Inc. vol. 47.

[ref71] Lindahl J. R. Britton W. B. (2019). ‘I have this feeling of not really being here’: Buddhist meditation and changes in sense of self. J. Conscious. Stud. 26, 157–183.

[ref72] Maiese M. (2022). Autonomy, Enactivism, and mental disorder: A philosophical account. London: Routledge.

[ref73] Maj M. (2005). ‘Psychiatric comorbidity’: an artefact of current diagnostic systems? Br. J. Psychiatry 186, 182–184. doi: 10.1192/bjp.186.3.182, PMID: 15738496

[ref74] Matthewson J. Griffiths P. E. (2017). Biological criteria of disease: four ways of going wrong. J. Med. Philos. 42, 447–466. doi: 10.1093/jmp/jhx004 28475734

[ref75] Maxwell J. A. (2012). A realist approach for qualitative research. Thousand Oaks, California: Sage Publications Inc.

[ref76] McLean K. C. Syed M. Pasupathi M. Adler J. M. Dunlop W. L. Drustrup D.. (2020). The empirical structure of narrative identity: the initial big three. J. Pers. Soc. Psychol. 119, 920–944. doi: 10.1037/pspp0000247 30998044

[ref9001] Merleau-Ponty, Maurice. (2012). The Phenomenology of Perception, Trans. Donald Landes, New York & London: Routledge.

[ref77] Mezzich J. E. Salloum I. M. Cloninger C. R. Salvador-Carulla L. Kirmayer L. J. Banzato C. E.. (2010). Person-centred integrative diagnosis: conceptual bases and structural model. Can. J. Psychiatry 55, 701–708. doi: 10.1177/070674371005501103, PMID: 21070697

[ref78] Motta V. N. (2023). Loneliness: from absence of other to disruption of self. Topoi 42, 1143–1153. doi: 10.1007/s11245-023-09984-5

[ref79] Neustadter E. S. Fotopoulou A. Steinfeld M. Fineberg S. K. (2021). Mentalization and embodied selfhood in Borderline Personality Disorder. J. Conscious. Stud. 28, 126–157. 34987307 PMC7612160

[ref80] Newen A. (2018). The embodied self, the pattern theory of self, and the predictive mind. Front. Psychol. 9:2270. doi: 10.3389/fpsyg.2018.02270 30532721 PMC6265368

[ref81] Newen A. Fabry R. E. (2023). A pattern theory of scaffolding. Rev. Phil. Psych. doi: 10.1007/s13164-023-00720-x

[ref82] Nielsen K. (2020). What is mental disorder? Developing an embodied, embedded, and enactive psychopathology”, (PhD Thesis Online), New Zealand: Victoria University of Wellington.

[ref83] Nordgaard J. Sass L. A. Parnas J. (2013). The psychiatric interview: validity, structure and subjectivity. Eur. Arch. Psychiatry Clin. Neurosci. 1-12. doi: 10.1007/s00406-012-0366-2 PMC3668119 23001456

[ref84] Paris J. (2013). The intelligent Clinician’s guide to the DSM-5. Oxford: Oxford University Press.

[ref85] Paris J. Kirmayer L. J. (2016). The National Institute of Mental Health research domain criteria: a bridge too far. J. Nerv. Ment. Dis. 204, 26–32. doi: 10.1097/NMD.0000000000000435 26704462

[ref86] Parnas J. Gallagher S. (2015). “Phenomenology and the interpretation of psychopathological experience” in Revisioning psychiatry integrating biological, clinical and cultural perspectives. eds. Kirmayer L. J. Lemelson R. Cummings C. (Cambridge: Cambridge University Press), 65–80.

[ref87] Parnas J. Møller P. Kircher T. Thalbitzer J. Jansson L. Handest P.. (2005). EASE: examination of anomalous self-experience. Psychopathology 38, 236–258. doi: 10.1159/000088441 16179811

[ref88] Parnas J. Sass L. A. Zahavi D. (2013). Rediscovering psychopathology: the epistemology and phenomenology of the psychiatric object. Schizophr. Bull. 39, 270–277. doi: 10.1093/schbul/sbs153 23267191 PMC3576163

[ref89] Petitmengin C. (2006). Describing one’s subjective experience in the second person: an interview method for the science of consciousness. Phenomenol. Cogn. Sci. 5, 229–269. doi: 10.1007/s11097-006-9022-2

[ref90] Petitmengin C. Remillieux A. Valenzuela-Moguillansky C. (2019). Discovering the structures of lived experience. Phenomenol. Cogn. Sci. 18, 691–730. doi: 10.1007/s11097-018-9597-4

[ref91] Pienkos E. (2020). Schizophrenia in the world: arguments for a contextual phenomenology of psychopathology. J. Phenomenol. Psychol. 51, 184–206. doi: 10.1163/15691624-12341377

[ref92] Pienkos E. Englebert J. Feyaerts J. Ritunnano R. Sass L. (2023). Situating phenomenological psychopathology. Front. Psychol. 14, 1–3. doi: 10.3389/fpsyg.2023.1204174 PMC10486011 37691804

[ref93] Pilgrim D. Bentall R. (1999). The medicalisation of misery: a critical realist analysis of the concept of depression. J. Ment. Health 8, 261–274. doi: 10.1080/09638239917427

[ref94] Porcher J. (2023). Hermeneutical injustice in the attribution of psychotic symptoms with religious content. Phil. Psychiatry Psychol. doi: 10.13140/RG.2.2.24356.04480

[ref95] Rasmussen A. R. Stephensen H. Parnas J. (2018). EAFI: examination of anomalous fantasy and imagination. Psychopathology 51, 216–226. doi: 10.1159/000488464, PMID: 29758549 PMC6106139

[ref96] Read J. Bentall R. Fosse R. (2009). Time to abandon the Bio-Bio-bio model of psychosis: exploring the epigenetic and psychological mechanisms by which adverse life events lead to psychotic symptoms. Epidemiol. Psychiatr. Sci. 18, 299–310. doi: 10.1017/S1121189X00000257, PMID: 20170043

[ref97] Reed G. M. Correia J. M. Esparza S. Saxena S. Maj M. (2011). The WPA-WHO global survey of psychiatrists’ attitudes towards mental disorders classification. World Psychiatry 10, 118–131. doi: 10.1002/j.2051-5545.2011.tb00034.x 21633689 PMC3105474

[ref98] Reed G. M. First M. B. Kogan C. S. Hyman S. E. Gureje O. Gaebel W.. (2019). Innovations and changes in the ICD-11 classification of mental, behaviourial and neurodevelopmental disorders. World Psychiatry 18, 3–19. doi: 10.1002/wps.20611 30600616 PMC6313247

[ref99] Riessman C. K. (2008). Narrative methods for the human sciences. Thousand Oaks, California: Sage Publications, Inc.

[ref100] Ritunnano R. (2022). Overcoming hermeneutical injustice in mental health: a role for critical phenomenology. J. Br. Soc. Phenomenol. 53, 243–260. doi: 10.1080/00071773.2022.2031234

[ref101] Rose D. Rose N. (2023). Is ‘another’psychiatry possible? Psychol. Med. 53, 46–54. doi: 10.1017/S003329172200383X 36628566

[ref102] Russell J. L. (2023). Problems for enactive psychiatry as a practical framework. Philos. Psychol. 36, 1458–1481. doi: 10.1080/09515089.2023.2174423

[ref103] Sass L. Pienkos E. Skodlar B. Stanghellini G. Fuchs T. Parnas J.. (2017). EAWE; examination of anomalous world experience. Psychopathology 50, 10–54. doi: 10.1159/000454928 28268224

[ref104] Scharfstein S. S. (2005). Big pharma and American psychiatry: the good, the bad, and the ugly. Psychiatric news. Amer. Psychiatry Assoc. 40, 3–4. doi: 10.1176/pn.40.16.00400003

[ref105] Scheffer M. Bockting C. L. Borsboom D. Cools R. Delecroix C. Hartmann J. A.. (2024). Dynamical systems view of psychiatric disorders-practical implications: a review. JAMA Psychiatry 81:624. doi: 10.1001/jamapsychiatry.2024.0228 38568618

[ref106] Seidman I. (2006). Interviewing as qualitative research: A guide for researchers in education and the social sciences. New York: Teachers College Press.

[ref107] Spencer L. J. (2023). (2023). Hermeneutical injustice and unworlding in psychopathology. Philos. Psychol. 36, 1300–1325. doi: 10.1080/09515089.2023.2166821 38013682 PMC10545401

[ref108] Spencer L. Broome M. (2023). The epistemic harms of empathy in phenomenological psychopathology. Phenomenol. Cogn. Sci. doi: 10.1007/s11097-023-09930-1 PMC12886269 41675301

[ref109] Stanghellini G. Aragona M. Gilardi L. Ritunnano R. (2022b). The person’s position-taking in the shaping of schizophrenic phenomena. Philos. Psychol. 36, 1261–1286. doi: 10.1080/09515089.2022.2144192

[ref110] Stanghellini G. Broome M. R. (2014). Psychopathology as the basic science of psychiatry. Br. J. Psychiatry 205, 169–170. doi: 10.1192/bjp.bp.113.138974 25179621

[ref111] Stanghellini G. Broome M. R. Raballo A. Fernandez A. V. Fusar-Poli P. Rosfort R. (2019). The Oxford handbook of phenomenological psychopathology. Oxford: Oxford University Press.

[ref112] Stanghellini G. Mancini M. Fernandez A. V. Moskalewicz M. Pompili M. Ballerini M. (2022a). Transdiagnostic assessment of temporal experience (TATE) a tool for assessing abnormal time experiences. Phenomenol. Cogn. Sci. 21, 73–95. doi: 10.1007/s11097-021-09795-2

[ref113] Stanier J. (2022). An introduction to engaged phenomenology. J. Br. Soc. Phenomenol. 53, 226–242. doi: 10.1080/00071773.2022.2081533 PMC9255638 35813180

[ref114] Stern L. Kirmayer L. J. (2004). Knowledge structures in illness narratives: development and reliability of a coding scheme. Transcult. Psychiatry 41, 130–142. doi: 10.1177/1363461504041358 15171211

[ref115] Tekin Ş. (2022). Participatory interactive objectivity in psychiatry. Philos. Sci. 89, 1166–1175. doi: 10.1017/psa.2022.47

[ref116] Wakefield J. C. (2007). The concept of mental disorder: diagnostic implications of the harmful dysfunction analysis. World Psychiatry 6, 149–156., PMID: 18188432 PMC2174594

[ref9002] Weinberger A. H. Smith P. H. Allen S. S. Cosgrove K. P. Saladin M. E. Gray K. M.. (2015). Systematic and meta-analytic review of research examining the impact of menstrual cycle phase and ovarian hormones on smoking and cessation. Nicotine Tob Res. 17:407–21. doi: 10.1093/ntr/ntu249 25762750 PMC4429881

[ref117] Windell D. L. Norman R. Lal S. Malla A. (2015). Subjective experiences of illness recovery in individuals treated for first-episode psychosis. Soc. Psychiatry Psychiatr. Epidemiol. 50, 1069–1077. doi: 10.1007/s00127-014-1006-x 25549829

[ref118] Zahavi D. (2020). The practice of phenomenology: The case of Max van Manen. Nurs Philos 21:e 12276. doi: 10.1111/nup.12276 31441216

[ref119] Zawadzki P. (2022). Pattern theory of self and situating moral aspects: the need to include authenticity, autonomy and responsibility in understanding the effects of deep brain stimulation. Phenomenol. Cogn. Sci. 21, 559–582. doi: 10.1007/s11097-020-09708-9

